# Can perioperative hemodilution be monitored with non-invasive measurement of blood hemoglobin?

**DOI:** 10.1186/s12871-021-01351-4

**Published:** 2021-05-06

**Authors:** Robert G. Hahn, Patrick Y. Wuethrich, Joachim H. Zdolsek

**Affiliations:** 1 Research Unit, Södertälje Hospital, Södertälje, and Karolinska Institutet at Danderyds Hospital (KIDS), Stockholm, Sweden; 2 Department of Anaesthesiology and Pain Medicine, Inselspital, Bern University Hospital, University of Bern, CH-3010 Berne, Switzerland; 3 Department of Anesthesiology and Intensive Care, and Department of Medical and Health Sciences, Linköping University, Linköping, Sweden

**Keywords:** Pulse oximetry, Hemoglobin, Non-invasive measurement, Point of care tests

## Abstract

**Background:**

Trends in non-invasive measurements of blood hemoglobin (Hb) may be useful for identifying the need for transfusion in the perioperative period.

**Methods:**

Crystalloid fluid (5–20 mL/kg) was administered intravenously or by mouth to 30 volunteers and 33 surgical patients in five non-randomized clinical studies where Hb was measured on 915 occasions by non-invasive (Radical-7™) and invasive methodology. The hemodilution curves were compared by volume kinetic analysis and linear regression, with the slope and scattering of the data as key outcome measures.

**Results:**

The slope was 1.0, indicating unity between the two modes of measuring Hb when crystalloid fluid was infused in volunteers; however, only 40–45% of the variability in the non-invasive Hb could be explained by the invasive Hb. Patients undergoing major surgery, who showed the most pronounced hemodilution (median 24 g/L); non-invasive Hb explained 72% of the variability but indicated only half the magnitude of the invasive Hb changes (slope 0.48, *P* < 0.001 versus the volunteers). Simulations based on volume kinetic parameters from the volunteers showed 25% less plasma volume expansion after infusion when based on non-invasive as compared to invasive Hb, while no difference was found during infusion.

**Conclusions:**

In volunteers the non-invasive Hb had good accuracy (low bias) but poor precision. In surgical patients the non-invasive Hb had good precision but systematically underestimated the hemodilution. Despite severe limitations, the non-invasive technology can be used to follow Hb trends during surgery if supported by occasional invasive measurements to assure acceptable quality of the hemodilution curve.

**Trial registrations:**

ControlledTrials.gov NCT01195025, NCT01062776, NCT01458678, NCT03848507, and NCT01360333 on September 3, 2010, February 4, 2010, October 25, 2011, February 20, 2019, and May 25, 2011, respectively.

**Supplementary Information:**

The online version contains supplementary material available at 10.1186/s12871-021-01351-4.

## Background

The measurement of the blood hemoglobin (Hb) concentration is a guide that determines when transfusion of erythrocytes should be initiated. Sporadic measurement of Hb is often performed during major surgery to evaluate when the Hb value corresponding to the “transfusion trigger” is reached. Continuous measurement of Hb would therefore be of substantial value to refine these decisions. For this purpose, non-invasive Hb analysis has been commercially available for about a decade in the form of the multi-wavelength pulse oximeter Radical-7™ from Masimo Inc.

Many studies have performed point-wise comparisons between non-invasive and invasive Hb [1–13], and the main problem with the non-invasive technology is its poor precision. Several studies show good accuracy but 95% limits of agreement with a range of 40 g/L or more [3–9], which makes non-invasive Hb measurements difficult to rely on.

A more essential clinical question is whether the changes in Hb agree over time in an individual patient. If this is the case, the pulse oximeter could be calibrated with the known invasive Hb when surgery starts and then be used to indicate relative changes. To study this possibility, we reviewed the linearity between changes in Hb level as given by non-invasive and invasive Hb monitoring, using data from five cohorts where the Hb level had shifted due to fluid therapy and/or hemorrhage.

The aim of the present report was to determine the reliability of the non-invasive Hb for tracking changes in invasive Hb. Our hypothesis was that the non-invasive alternative is a useful index of Hb changes. This issue was studied by direct comparisons of Hb trends and also by assessing kinetic analyses of plasma volume shifts based on either mode of Hb measurement.

## Methods

Data from five case non-randomized clinical trials where the Hb concentration was altered by intravenous or oral administration of crystalloid fluid with and without hemorrhage was reviewed. All studies were approved by the appropriate local Ethics committee and were registered at ClinicalTrials.gov before any patient was recruited. This report adheres to the CONSORT guidelines.

### Procedures

The five series comprised only euhydrated subjects.

- Study 1 included the infusions of 25 mL kg^− 1^ of Ringer’s solution given 10 healthy male volunteers [11].
- Study 2 evaluated the ability of volume kinetics to detect deliberately induced dehydration; here, only the 10 control infusions of 10 mL kg^− 1^ of Ringer’s were used [12].
- Study 3 used repeated Hb sampling during a short infusion of 5 mL kg^− 1^ of Ringer’s in the morning before surgery; the cohort originally included 30 patients, but only 10 of them were randomly selected for inclusion in the present study to maintain balance between volunteers and surgical patients [13].
- Study 4 included 23 patients who underwent open radical cystectomy and urinary diversion under general anesthesia combined with thoracic epidural analgesia. Baseline fluid maintenance of 1 mL kg^− 1^ h^− 1^ of Ringer’s until the end of the removal of the bladder and thereafter increased to 3 mL kg^− 1^ h^− 1^ of Ringer’s until the end of surgery. A fixed dose of 20% albumin solution (3 mL kg^− 1^; Albumin CSL 20%, CSL Behring, Bern, Switzerland) was given over 30 min until removal of the bladder (expected blood loss of > 500 mL) was started. Blood loss was replaced with the Ringer solution in a 1:1 ratio. A supportive administration of norepinephrine, starting at a rate of starting at 30 ng kg^− 1^ min^− 1^ (to maintain the mean arterial blood pressure at > 65 mmHg) and mini fluid challenges of 100 mL of Ringer’s (to maintain the hemodynamics pulse pressure variation > 10), until normovolemia was restored [14]. One unit of erythrocytes was transfused, but this was administered after data collection had been completed.
- Study 5 comprised healthy volunteers who ingested 500 mL of isotonic saline by mouth [15].

Table 1 shows the details and characteristics of the five cohorts, which have been published with other study questions. Precise inclusion and exclusion criteria were given for the respective studies [11–15], but subjects with clinically apparent hemodynamic, hepatic, renal, or pulmonary impairment were not included in any of them.

**Table 1:** Demographics and characteristics of the studied patient groups

	Study 1	Study 2	Study 3	Study 4	Study 5
Subjects	Volunteers	Volunteers	Before major surgery	During open cystectomy	Volunteers
N	10	10	10	23	10
Males / females	10 / 0	10 / 0	7 / 3	19 / 4	7 / 3
Age, years	22 (18–28)	22 (19–37)	64 (29–82)	69 (67–75)	33 (10)
Body weight, kg	79 (65–101)	80 (75–100)	79 (70–87)	87 (64–90)	77 (13)
Infused	Ringer’s acetate	Ringer’s acetate	Ringer’s acetate	20% albumin + Ringer’s lactate	Isotonic saline
Amount, time	20 mL/kg	10 ml/kg	5 mL/kg	3 mL/kg + 2100 mL	500 mL
Infusion time (min)	30	20	15	300	2–3 min
Hemorrhage (mL)	0	0	0	950 (700–1225)	0
Sampling points (N)	25	16	9	14	10
Duration (min)	180	120	70	300	120
Reference	11, 16	2, 12	13	14	15
Ethics approval, city	Stockholm, SE	Linköping, SE	Linköping, SE	Bern, CH	Linköping SE
Ethics approval, Nr	2009/1091–31/2	M114–09	2011/101–31	2018–02351	2010/241–31
ClinicalTrials.gov	NCT01195025	NCT01062776	NCT01458678	NCT03848507	NCT01360333

Repeated non-invasive measurements of Hb and the perfusion index in the hand were obtained from a pulse oximeter (Radical 7, Masimo Corp., Irvine, CA). At the same points in time, a blood sample was obtained for later measurement of the Hb concentration at the hospital’s official chemical laboratory with a coefficient of variation of approximately 1.0%. Blood gas machines were not used.

### Linear relationships

Linear relationships were evaluated by comparing non-invasive and invasive Hb on a regression plot. The **scatter** was used as the measure of precision and expressed as the coefficient of determination (r^2^) of the regression line. The coefficient of determination is the fraction of the variability of y (non-invasive Hb) that can be explained by x (invasive Hb). A high r^2^ is desirable, as it implies that data scattering around the regression line is small.

Whether the non-invasive decreases as much as the invasive Hb during hemodilution is a different issue. We use the **slope**, i.e. the steepness, of the regression line between these two variables to express the accuracy of a series of non-invasive measurements in the same subject. A slope lower than 1.0 means that the numerical change in non-invasive Hb is less than that for the invasive Hb. Values higher than 1.0 denote the opposite relationship.

### Volume kinetics

The kinetics of the infusion fluid was studied by a mixed model approach on the non-invasive and invasive data separately. The first three were included while the fourth was excluded due to the large hemorrhage (median 950 mL) and the use of two infusion fluids. The fifth was also excluded because the oral ingestion of fluid would necessitate the use of a structurally different kinetic model than was used for the other studies.

The model had two dependent variables, the total urinary excretion and frequently measured plasma dilution. The latter was derived as the hemodilution divided by (1–baseline hematocrit). In turn, the hemodilution had been obtained as (Hb_o_–Hb)/Hb, in which Hb_o_ is the baseline value and Hb denotes measurements taken at a later time.

The model has been created to reflect body physiology. *V*_c_ is thought to correspond to the plasma from which distribution and redistribution occurs to an extravascular space, *V*_t_. Hence, a two-volume kinetic model with rate constants for distribution and re-distribution (*k*_12_ and *k*_21_), one route of elimination (*k*_10_), and one scaling factor between dilution and volume (*V*_c_, central volume) was fitted to two dependent variables in all experiments on a single occasion, using the Phoenix software for nonlinear mixed effects, version 1.3 (NLME, Pharsight, St. Louis, MO). All flows are proportional to the value of the rate constant to the volume expansion of a fluid space, being *V*_c_ for *k*_12_ and *k*_10_, and *V*_t_ for *k*_21_. The estimate of *k*_10_ was stabilized by the measured urinary excretion, as described elsewhere [11–13]. The search routine used was the First-Order Conditional Estimation Extended Least Squares (FOCE ELS).

### Statistics

Data are reported as the median (25th to 75th percentile) and kinetic parameters as the best estimate and 95% confidence interval. The agreement between the two modes of measuring Hb was illustrated by Bland-Altman plots that showed the mean difference (the agreement) and the associated 95% limits of agreement (i.e., ± 2 standard deviations of the differences). Point-wise comparisons been non-invasive and invasive Hb measurements has previously been reported from three of the five case series [11–13].

Relationships were studied by simple linear regression, where r^2^ = the coefficient of determination (the “scatter” of the data).

The number of experiments was set at 60, which was felt to be an appropriate number for outlining the relationship between non-invasive and invasive Hb. Power analysis was applied in the respective studies. *P* < 0.05 was statistically significant.

## Results

**Fig. 1:**
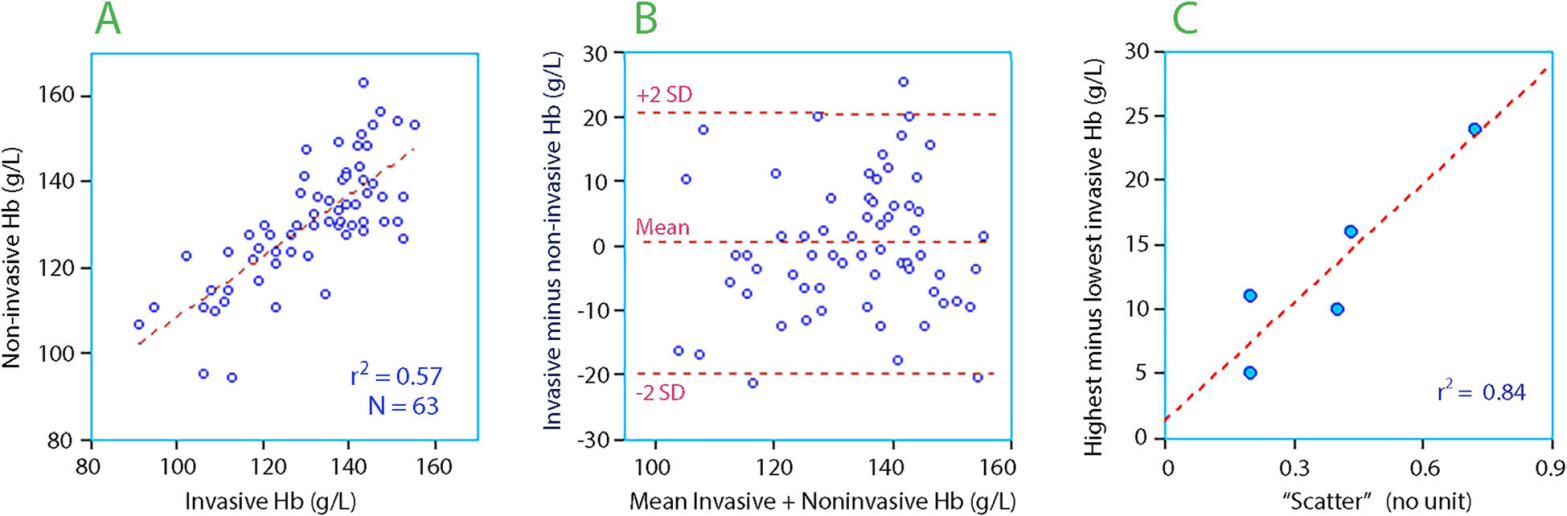
**a** The invasive versus non-invasive Hb concentration at the onset of the infusions. **b** Bland-Altman plot of the data shown in the previous subplot. **c** Relationship between the median “scatter” around the regression lines for each of the five groups, i.e. the coefficient of determination

At baseline, a statistically significant correlation was observed between the invasive and non-invasive measurement of Hb (Fig. 1a). The mean difference was only + 0.5 g L^− 1^, but the 95% limits of agreement ranged from − 20 to + 21 g/L (Fig. 1b).

### Maximum and minimum Hb

The highest invasive Hb measured in the 63 patients was 136 (121–144) g L^− 1^ and the lowest 117 (107–130) g L^− 1^. For the non-invasive Hb, these values were 131 (123–141) g L^− 1^ and 117 (105–126) g L^− 1^, respectively (Table 2). The difference between highest and lowest was quite similar for the two modes of measuring Hb, at 14 (10–21) vs. 15 (11–20) g L^− 1^. Hb was ≤100 g L^− 1^ in 90 of the 915 invasive measurements (10%).

**Table 2:** Comparisons between non-invasive and invasive Hb

	Study 1	Study 2	Study 3	Study 4	Study 5
Invasive Hb					
Highest Hb (g/L)	144 (142–145)	138 (132–144)	123 (113–136)	129 (116–140)	138 (132–149)
Lowest Hb (g/L)	127 (124–130)	128 (122–134)	114 (104–121)	107 (97–113)	134 (126–136)
Highest – lowest Hb (g/L)	16 (16–19)	10 (9–11)	11 (9–14)	24 (16–28)	5 (5–6)
Maximum change (%)	13 (13–15)	8 (7–9)	10 (7–12)	22 (14–27)	4 (3–5)
Patient mean dilution (%)	7.0 (5.2–7.8)	3.9 (2.9–5.4)	4.2 (3.4–6.7)	11.5 (6.7–14.3)	2.9 (1.4–2.7)
Non-invasive Hb					
Highest SpHb (g/L)	139 (131–149)	134 (130–141)	126 (111–137)	123 (117–136)	134 (130–150)
Lowest SpHb (g/L)	118 (109–126)	117 (111–122)	108 (99–120)	112 (96–124)	126 (123–139)
Perfusion index (%)	5.2 (3.0–7.5)	7.9 (5.7–8.9)	5.0 (2.5–5.7)	3.2 (2.1–3.5)	4.1 (2.6–6.4)
Highest – lowest Hb (g/L)	21 (19–25)	19 (14–20)	15 (11–19)	13 (7–23)	10 (8–11)
Maximum change (%))	17 (15–24)	15 (12–18)	13 (9–16)	11 (6–21)	8 (7–8)
Patient mean dilution (%)	8.6 (6.5–10.8)	5.3 (2.0–7.8)	2.4 (0.4–5.2)	4.3 (3.5–8.9)	0.2 ((−0.8)–0.7)
Comparisons					
Scatter (r^2^) Hb values	0.43 (0.22–0.61)	0.40 (0.25–0.63)	0.20 (0.05–0.44)	0.72 (0.48–0.81)	0.20 (0.07–0.37)
Slope Hb values	0.95 (0.79–1.12)	1.04 (0.90–1.39)	0.46 (0.37–0.77)	0.49 (0.36–0.72)	0.16 ((−0.72)–0.70)
Slope Hb dilution	0.96 (0.75–1.14)	1.06 (0.72–1.73)	0.43 (0.32–0.78)	0.38 (0.29–0.81)	0.16 ((−0.72)–0.49)
Difference Hb dilution Invasive - non-invasive	−1.1 ((−3.6)–0.2)	−1.1 ((3.4)–1.9)	1.2 ((− 0.1)–6.0)	6.0 (2.4–9.7)	1.6 ((− 0.1)–3.6)

Data is the median (25th–75th percentile range)

In the subsequent presentation, we refrain from making point-wise comparison between the two technologies but focus on the linearity between their relative changes in a series of measurements in the same subject when Hb is deliberately changed. The results are expressed as the scatter (precision) and slope (accuracy) of non-invasive Hb to indicate the invasive Hb.

### Scattering

Overall, 46 (22–72)% of the changes of non-invasive Hb could be explained by changes in invasive Hb, i.e. the median “scatter” (r^2^) was 0.46. However, variability between the studies was considerable; the data were most closely assembled around the regression line during major surgery, r^2^ being 0.72 (Table 2).

As a rule, the scatter decreased with the Hb range in each series; a greater difference between the highest and lowest invasive Hb led to a higher r^2^ for the relationship between the two variables (Fig. 1c).

### Slope

**Fig. 2:**
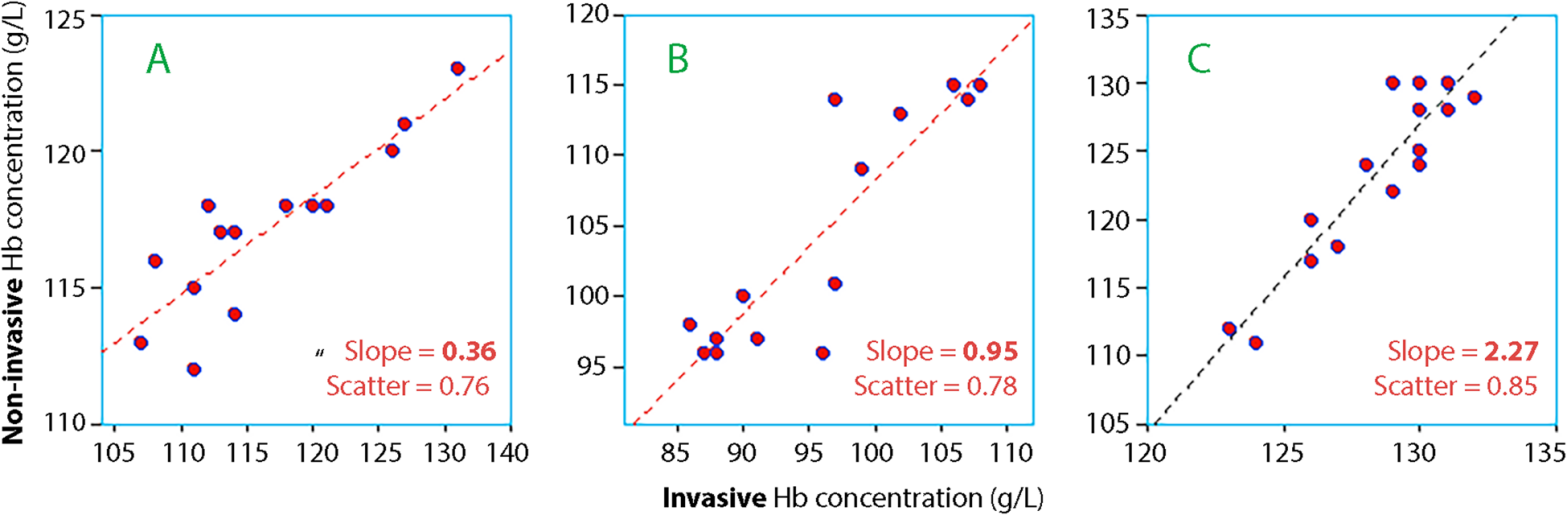
Three experiments with different slopes. Distances between the marks are set equal in each subplot to facilitate comparison. **a** Flat slope. The non-invasive Hb changes much less than the invasive Hb. **b** Normal slope. The rise in non-invasive Hb is similar to the rise of the invasive Hb. **c** Steep slope. Greater increase in non-invasive Hb than for the invasive technology

The regression slope shows how much of a change in invasive Hb is indicated by non-invasive Hb on the average during an entire experiment. The non-invasive Hb could either underestimate (weak slope, < 1.0), predict well (good slope = 1.0), or overestimate (steep slope, > 1.0), respectively, the hemodilution given by the invasive Hb. Figure 2 shows three individual experiments where the slope factor for the regression was weak, good, and steep.

Overall, the slope factor was 0.57 (0.33–1.01) but it differed greatly depending on the presence of surgical stress, being 1.00 (0.80–1.14) in the volunteer studies and 0.48 (0.37–0.73) in in association with surgical stress (studies 3 and 4; *P* < 0.006).

In Study 4, the surgical hemorrhage volume was 950 (780–1225) mL and did not correlate with the data scattering or with the slope factor.

### Hemodilution

**Fig. 3:**
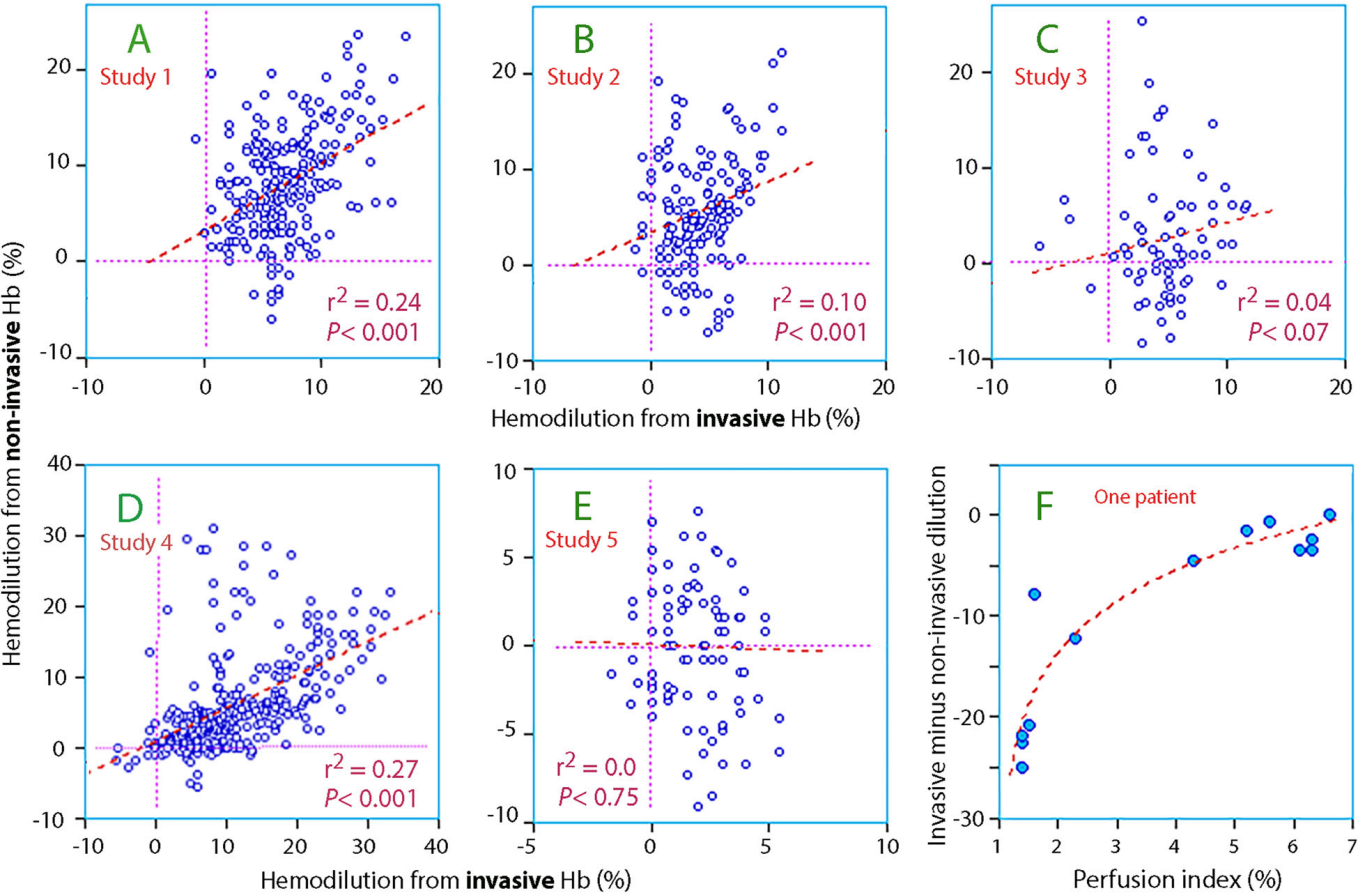
Relationship between the hemodilution as calculated from the invasive versus the non-invasive Hb measurements in the five studies (**a** to **e**) and the correlation between perfusion index and the deviation between the two modes of measuring Hb in a single patient from Study 4

Figure 3 compares all invasive and non-invasive hemodilution measurements in each sub-study. The patient mean hemodilution was 6.0 (3.5–10.3)% for the invasive and 4.9 (1.3–8.0)% for the non-invasive technologies (*P* < 0.01).

The non-invasive Hb underestimated the hemodilution given by the invasive Hb in the surgical stress studies, but not the volunteer studies; the difference between the patient mean hemodilution, as given by invasive and non-invasive Hb, was − 0.1 (− 2.4 to + 2.5)% in the volunteer studies but + 4.2 (1.0–8.3)% in the studies associated with surgical stress (*P* < 0.001).

**Fig. 4:**
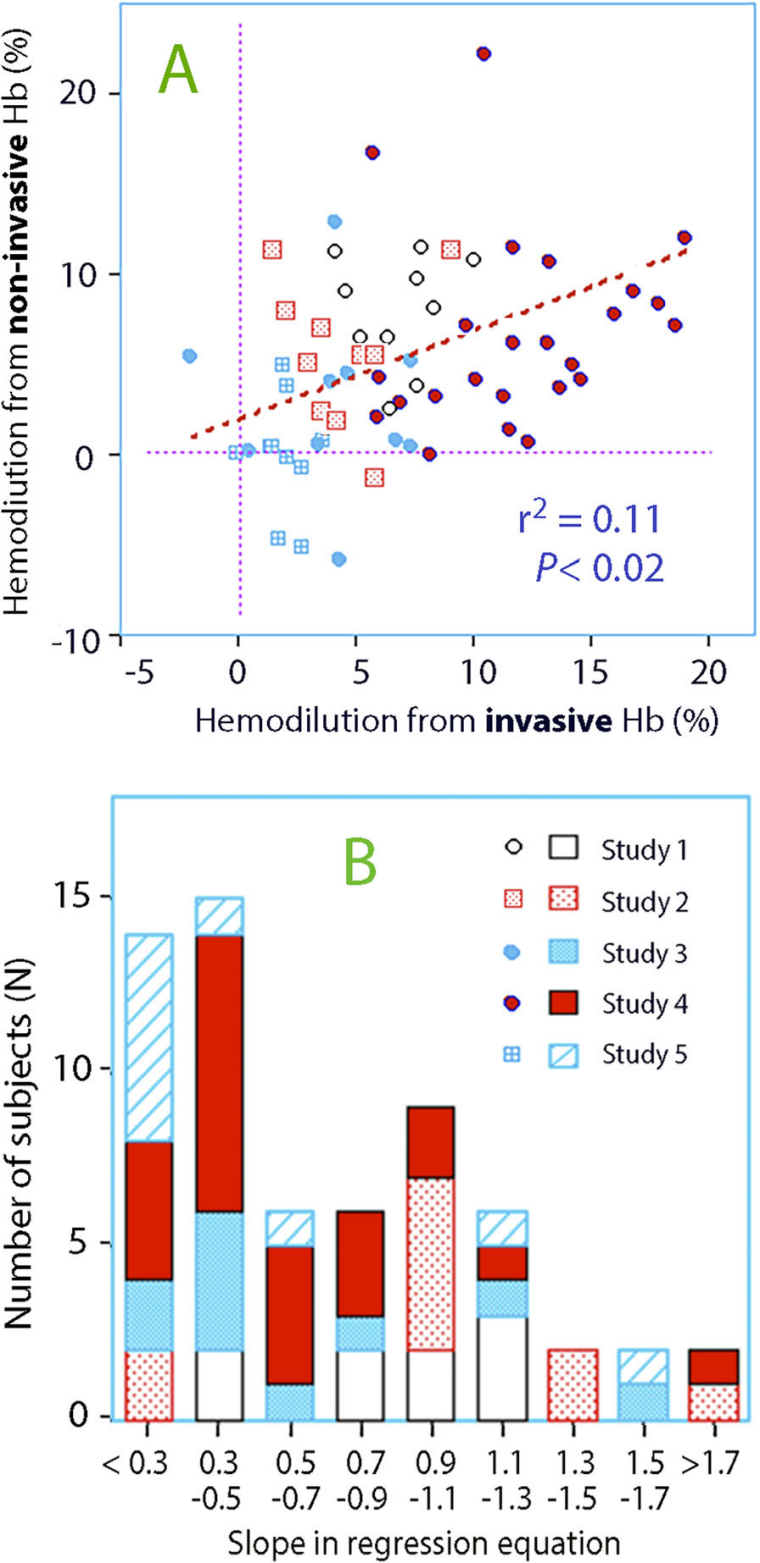
**a** Hemodilution as obtained by the invasive Hb versus the non-invasive Hb for each patient. **b** Distribution of slope factors in the five studies

Figure 4a compares, in each subject, the relationship between the mean hemodilution as given by invasive and non-invasive Hb.

Figure 4b shows the distribution of subjects between ranges of slope factors.

### Low perfusion index

**Fig. 5:**
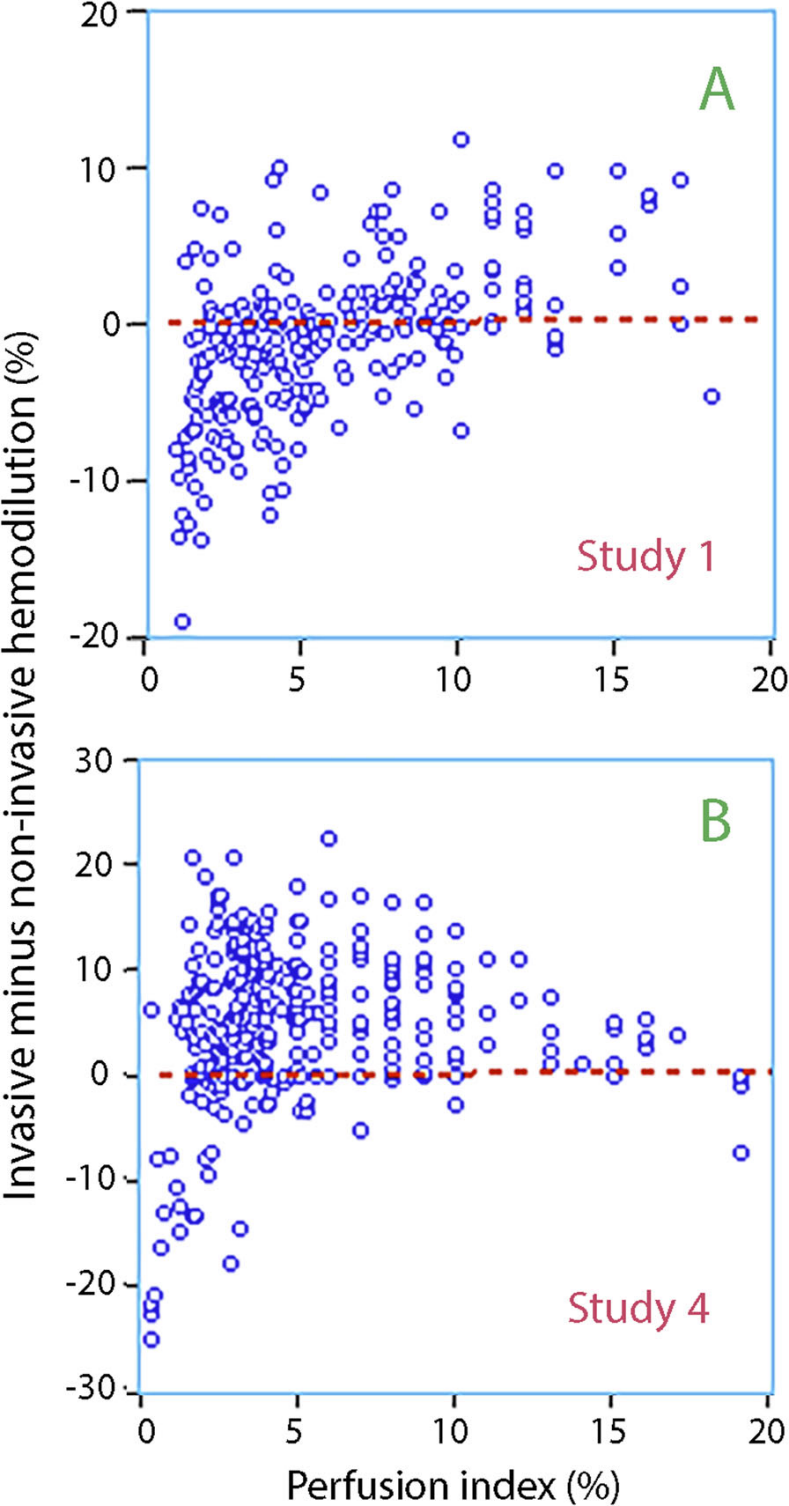
The perfusion index versus the difference in hemodilution as obtained by the two modes of measuring Hb in (**a**) Study 1 and (**b**) Study 4. These were the only cohorts in which the perfusion index occasionally dropped below 1.0

Only 15 of the 915 points of measurement (1.6%) were performed in the presence of a perfusion index of 1.0 or lower, and these low values occasionally distorted the data in Study 1 and 4 (Fig. 3f, Fig. 5).

### Volume kinetics

**Fig. 6:**
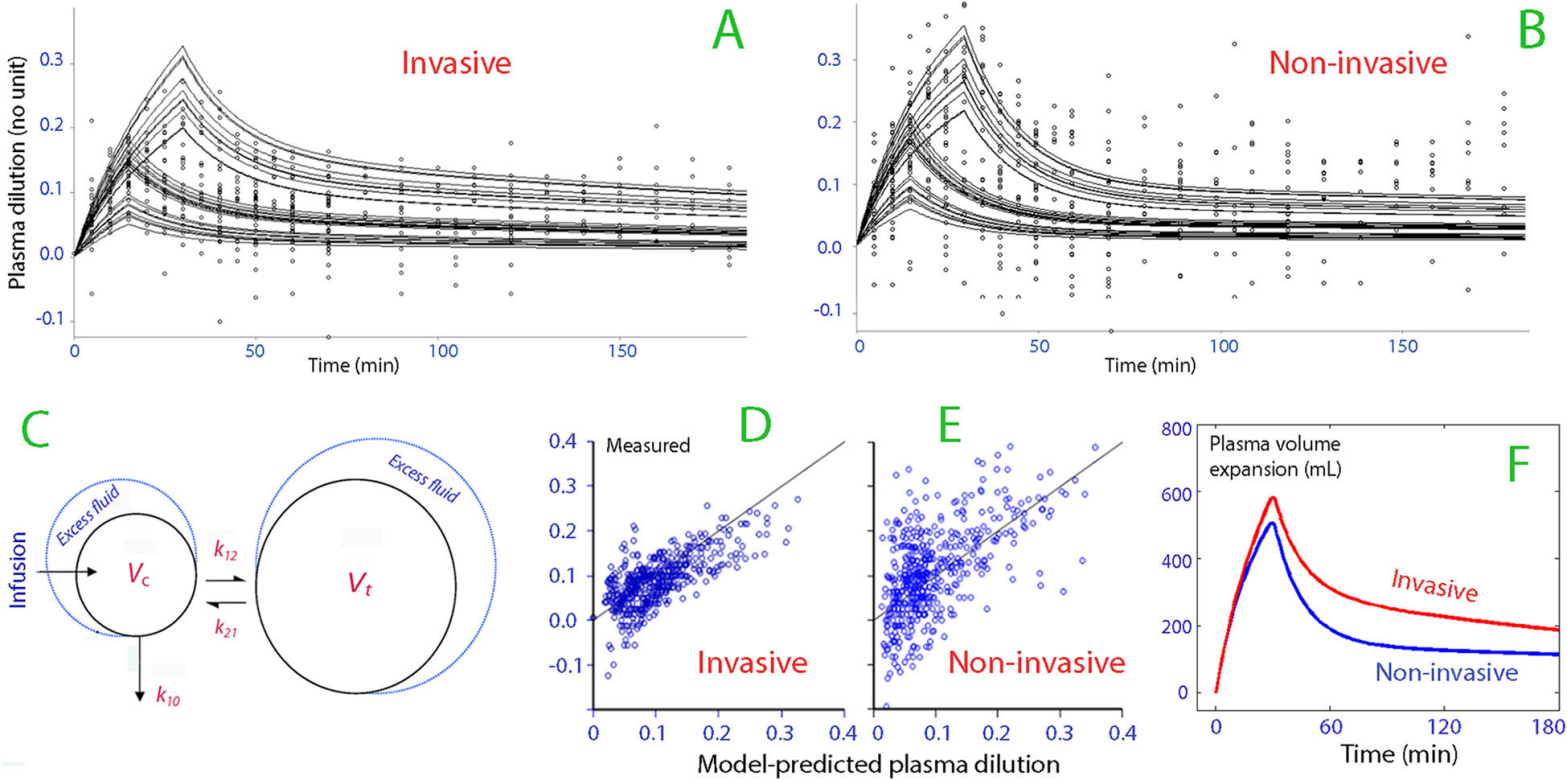
Volume kinetics. Curve-fitting of the Hb data from Studies 1–3 as obtained by the (**a**) invasive and (**b**) non-invasive measurements. **c** Schematic drawing of the kinetic model. **d** The model-predicted versus the measured plasma dilution as obtained with the (**d**) invasive and (**e**) non-invasive Hb. **f** Simulation of the volume expansion of the central body fluid space (the plasma) when 1 L of Ringer’s acetate is infused over 30 min

Fitting the two-volume kinetic model to the data on plasma dilution and urinary excretion was successful for both the invasive and the non-invasive methods (Fig. 6a-c). The precision of the kinetic parameters was higher for the invasive Hb than for the non-invasive Hb (Table 3), and more outliers became apparent when the kinetic parameters were used to re-create the dependent variables (Fig. 6d and e).

**Table 3:** Population kinetic parameters

Kinetic parameter	Measurement route	Best estimate	95% CI	CV%
*V*_c_ (L)	Invasive	3.91	3.27–4.56	8.5
	Non-invasive	3.11	2.51–3.71	9.8
*k*_12_ (10^− 3^ min^− 1^)	Invasive	38.3	30.9–46.0	10.0
	Non-invasive	47.0	22.0–72.0	27.1
*k*_21_ (10^−3^ min^− 1^)	Invasive	23.5	14.0–32.9	20.5
	Non-invasive	11.2	(−18.5)–41.0	135.2
*k*_10_ (10^−3^ min^−1^)	Invasive	9.1	3.7–14.3	29.8
	Non-invasive	9.4	2.7–16.1	36.5

Data are based on 30 infusion experiments (Studies 1, 2 and 3)

*CI* confidence interval, *CV* between-subject coefficient of variation

The best estimates of all parameters, as shown in Table 3, were used to simulate the outcome of fictious infusions. Those plots showed that the simulated expansion of the plasma volume was smaller when the non-invasive Hb was used — a difference that can be explained by the combination of higher *k*_12_ and lower *k*_21_ (Fig. 6f).

## Discussion

The five evaluated cohorts indicate the possibilities and limits of measuring Hb non-invasively. Overall, the difference between the highest and lowest Hb values was quite similar between the two technologies but, as shown many times before, there were wide limits of agreement at the baseline. Nevertheless, changes in Hb measured by the pulse oximeter often followed quite well the trends yielded by invasive measurement. However, this was not a universal finding. The non-invasive technique showed good precision but underestimated the magnitude of the invasive Hb changes in the presence of pre-surgical or surgical stress. By contrast, the precision was poor but the accuracy good in the volunteer studies.

### Surgical setting versus non-stressed volunteers

The clinical question of whether erythrocytes should be transfused was most real in Study 4 where measurements were performed during major surgery. The blood loss during the data collection amounted to almost 1 l, and the patients received both Ringer’s and 20% albumin. Norepinephrine infusion was used to maintain the arterial pressure. The hemodilution was twice as large as that seen in any of the other studies.

The non-invasive Hb followed the trends observed in the invasive Hb quite well, with an overall r^2^ as high as 0.72 despite occasional outliers that were sometimes due to a low perfusion index. One should note that 0.72 is the mean linearity from individual analysis of each patient, whereas the r^2^ yielded by point-wise comparisons based on the pooled data from all patients in Study 4 was considerably lower, only 0.27. Importantly, the magnitude of the hemodilution changes was underestimated, and it usually amounted to only half of the invasive value (Fig. 3d).

The underestimation of the hemodilution by the non-invasive approach is shared by Study 3, which was performed in patients awaiting major open abdominal surgery. The fact that the two clinical studies show the same pattern — namely, a too small hemodilution indicated by the non-invasive Hb and, hence, a low slope factor — suggests that adrenergic stress limits the magnitude of the Hb changes detected by the non-invasive technology, albeit without distorting the trend over time.

Studies 1 and 2 were both performed in a similar way in volunteers, but using different amounts of crystalloid fluid. Both studies had slopes with a median value close to 1.0, and the non-invasive hemodilution was approximately 20% greater than that obtained with the invasive Hb. Hence, the relationship between the two modes of measurement was opposite to that obtained in the perioperative setting, which supports the notion that data obtained in volunteers cannot be uncritically assumed to be valid in stressful situations.

By contrast, the performance of the non-invasive technology with oral administration of fluid was disappointing. Here, non-invasive measurement usually showed no change at all, or a trend opposite to that obtained by the invasive Hb. However, the average hemodilution obtained by invasive sampling averaged only 3%, which then seems too small to overcome the between-subject variability of the non-invasive Hb technique to any reasonable degree.

### Volume kinetics

Another issue is whether non-invasive monitoring can be used for the study of fluid population kinetics. For this purpose, the Hb data had to be converted to plasma dilution, which attains values that are approximately twice as large as the hemodilution. Previous studies have used non-invasive Hb for kinetic analysis in a two-stage approach, but with limited success [2, 12]. Here, all appropriate curves were analyzed in one single run, which offers a more stable output.

The analysis shown in Fig. 6 reveals that fitting the commonly used two-volume kinetic model to the present data produced more outliers and less precise estimates of the model parameters when based on the non-invasive as compared to the invasive technology. In our fictitious fluid experiment, the difference between the modes of measuring Hb became apparent during the distribution phase but not during infusion.

### Present report versus the literature

Many previous studies have examined the methodological bias by making point-wise comparisons between invasive and non-invasive measurements of Hb, usually without separating intra- and inter-individual differences. Some studies show a bias of 1–2% [1–4], while studies in cardiac and emergency care report underestimations of the invasive Hb of as much as 12–18 g L^− 1^ by the non-invasive technique [5–7]. These results support our observation that changes in non-invasive Hb become too small in stressed settings. The precision has consistently been poor, with 95% limits of agreement being in the range of 40–50 g L^− 1^ [3, 8], or even wider [4–7]. The conclusion in a meta-analysis of 32 studies from 2016 confirmed that the mean difference between the two modes of measuring Hb is small, while the range between the 95% limits of agreement is too wide to allow clinical decisions to be based on them [1–17].

Our review does not focus on point-wise comparisons; rather, it emphasizes a series of Hb changes, which we feel is more informative about the value of this monitoring in the perioperative setting. Here, hemodilution curves derived by the two modes of measuring Hb most often showed quite similar trends, but the many successful cases (high r^2^) were occasionally interrupted by curves showing poor agreement, so as to be misleading. Some of these were “switches” remain unexplained. The most stable indications were obtained in the surgical setting, where r^2^ was below 0.25 in only 2/23 patients, although the non-invasive Hb systematically underestimated the hemodilution. The scattering was aggravated in the presence of a very low perfusion index. By contrast, Chang et al. [10], found the accuracy of the Radical-7 to be good even when the perfusion index is below 1.0.

### Limitations

Limitations of this analysis include that the invasive Hb was measured in venous blood while the non-invasive method is believed to represent arterial or capillary Hb. However, the two sites of measurement should not differ much in terms of Hb concentration [18]. The five series of Hb experiments were collected under different conditions, which are described in detail elsewhere, but these differences do form the basis of the present conclusions [11–16]. Data were collected over a period of almost 10 years, during which the algorithm for calculating non-invasive Hb is claimed to have been improved. However, more recent work on Hb during spinal surgery and liver transplantation [10, 19] show almost identical accuracy and precision for the non-invasive Hb as we reported in 2010 [2]. These authors also studied the concordance between changes in invasive and non-invasive Hb during surgery [10, 19]. However, we chose to present our results differently, as we obtained data at very short intervals, usually only 5–10 min, and up to 25 times per experiment.

## Conclusions

Five series of Hb measurements showed that the linearity between invasive and non-invasive Hb data was strongest for the most pronounced hemodilution in surgical patients, albeit the hemodilution was underestimated. Data from volunteers yielded better accuracy but showed more scatter (poorer precision). Results were unreliable for hemodilution in the range of 3–4%. The non-invasive methodology has severe limitations, but Hb trends can be monitored relatively well during surgery to identify the need for erythrocyte transfusion if. However, we recommend that quality of the non-invasive Hb curve supported by timely invasive measurements.

## Supplementary Information

**Additional file 1.**

## Data Availability

The data used for the kinetic analysis is available as Additional file.xls (studies 1, 2 and 3). Data for Studies 4 and 5 are available from the corresponding author on request.

## Abbreviations

CI: Confidence interval
CV: Coefficient of variation
Hb: Hemoglobin
*k*_12_: Rate constant for fluid passing from the plasma to the extravascular fluid space
*k*_21_: Rate constant for fluid passing from the extravascular fluid space to the plasma
*k*_10_: Rate constant for fluid leaving the kinetic system as urine
L: Liter
mL: Milliliter
r^2^: Coefficient of determination (“scatter”)
SpHb: Pulse co-oximetry hemoglobin
*V*_c_: Size of central body fluid space
*V*_t_: Size of peripheral (extravascular) body fluid space
